# Neuropeptide Y and α-MSH Circadian Levels in Two Populations with Low Body Weight: Anorexia Nervosa and Constitutional Thinness

**DOI:** 10.1371/journal.pone.0122040

**Published:** 2015-03-23

**Authors:** Bogdan Galusca, Gaëtan Prévost, Natacha Germain, Isabelle Dubuc, Yiin Ling, Youssef Anouar, Bruno Estour, Nicolas Chartrel

**Affiliations:** 1 Department of Endocrinology, CHU de Saint-Etienne, Saint-Etienne, France; 2 Institut National de la Santé et de la Recherche Médicale (INSERM) Unité 982, Laboratory of Neuronal and Neuroendocrine Differentiation and Communication, Institute for Research and Innovation in Biomedecine (IRIB), University of Rouen, Mont-Saint-Aignan, France; CNRS, University of Strasbourg, FRANCE

## Abstract

**Context:**

Anorexia nervosa (AN) presents an adaptive appetite regulating profile including high levels of ghrelin and 26RFa (orexigenic) and low levels of leptin and PYY (anorexigenic). However, this adaptive mechanism is not effective in promoting food intake. The NPY/proopiomelanocortin (POMC) system plays a crucial role in the regulation of feeding behavior as NPY is the most potent orexigenic neuropeptide identified so far and as the POMC-derived peptide α-MSH drastically reduces food intake, and this peptidergic system has not been thoroughly studied in AN.

**Objective:**

The aim of the present study was thus to investigate whether a dysfunction of the NPY/POMC occurs in two populations with low body weight, AN and constitutional thinness (CT).

**Design and Settings:**

This was a cross-sectional study performed in an endocrinological unit and in an academic laboratory.

**Investigated Subjects:**

Three groups of age-matched young women were studied: 23 with AN (AN), 22 CT and 14 normal weight controls.

**Main Outcome Measures:**

Twelve-point circadian profiles of plasma NPY and α-MSH levels were measured in the three groups of investigated subjects.

**Results:**

No significant circadian variation of NPY was detected between the three groups. Plasma α-MSH levels were significantly lower in AN (*vs* controls) all over the day. The CT group, compared to controls, presented lower levels of α-MSH in the morning and the evening, and an important rise during lunchtime.

**Conclusion:**

In AN patients, the NPY system is not up-regulated under chronic undernutrition suggesting that this may play a role in the inability of anorectic women to adapt food intake to their energy demand. In contrast, low circadian α-MSH levels integrate the adaptive profile of appetite regulation of this disease. Finally, in CT women, the important α-MSH peak detected during lunchtime could explain why these patients are rapidly food satisfied.

## Introduction

Anorexia nervosa (AN) is characterized by dramatic decrease of food intake, important weight loss (BMI<17.5 kg/m^2^) and multiple endocrine changes [1]. Whatever the causal factors leading to AN, all anorectic patients are unable to adapt their feeding behavior to energy demand and costs, suggesting a possible dysfunction of hormones regulating appetite. We previously tested this hypothesis and found that plasma levels of anorexigenic hormones such as leptin and PYY were drastically decreased all over the day in AN patients [2, 3], whereas the orexigenic peptides such as ghrelin and 26RFa were highly increased during the nychtemeron [3–6]. These observations suggest the occurrence in AN patients of an adaptive mechanism to promote food intake and thus to counteract chronic undernutrition. However, the increase in orexigenic peptides and the decrease in anorexigenic hormones do not lead to a stimulation of food intake in AN, indicating a resistance to these feeding signals. The neuropeptide Y (NPY)/proopiomelanocortin (POMC) system plays a crucial role in the regulation of feeding behavior as NPY is the most potent orexigenic neuropeptide identified so far and as the POMC-derived peptide α-MSH drastically reduces food intake [7]. Surprisingly, data on an eventual dysregulation of the NPY/POMC system in AN are poorly documented and controversial [8–13].

With a BMI similar to that of AN girls, constitutionally thin women (CT) do not exhibit abnormal feeding behavior and caloric deficits, and most endocrine parameters are similar to controls, which make them different from AN patients [14, 15]. In CT, we described a completely different profile with normal plasma ghrelin [2, 3] and 26RFa [4], but slight increase in PYY [2] that could contribute to their inability to gain weight. To date the NPY/ POMC system was not assessed in CT.

In the present report we investigated an eventual dysfunction of the NPY/POMC system in AN by establishing the circadian profiles of plasma NPY and α-MSH levels. We also compared the plasma NPY and α-MSH levels of AN patients with those of control subjects and women with CT.

## Subjects and Methods

The present study was approved by the local Human Research Ethic Committee of the Saint-Etienne University Hospital Center (France), and all of the subjects gave a written informed consent for biological assessment.

### Subjects

The study included three groups of young Caucasian women (18–27 years): anorexia nervosa restrictive-type (AN), constitutionally thin (CT) and control subjects (C). The AN and CT groups were BMI matched.

Twenty three AN subjects were recruited among our in/out patient after the diagnosis of restrictive type anorexia nervosa was done. The study was performed during the first hospitalization of the patients, and before any therapeutic intervention. The subjects met the diagnostic criteria for AN of the *Diagnostic and Statistical Manual of Mental Disorders* [16], and had a BMI < 16.5 kg/m^2^. None of them used oral contraceptives and all of them exhibited secondary amenorrhea for more than six months.

Twenty two CT subjects were recruited among the patients evaluated for leanness, using the following criteria: BMI < 16.5 kg/m^2^, stable throughout the growth period, physiological menstruations without oral contraceptives and a stated desire for weight gain.

Fourteen normal weight controls (mean BMI between 19 and 25 kg/m^2^) without any eating disorders were also evaluated.

In CTs and controls, all data were collected during the follicular phase of cycle. None of the subjects had documented intense physical activity, chronic or congenital disease, and none of them were taking any medication. Preliminary estimation of physical activity with MONICA Optional Study of Physical Activity Questionnaire was performed in order to exclude subjects with intense physical activity [17].

### Samplings

Blood sampling was carried out during an inpatient period of 24 hours and all of the subjects had a sedentary lifestyle during the 24-hours sampling. After venous collection, blood samples were immediately centrifuged and plasmas were aliquoted and kept frozen at -80 C before the assays. NPY and α-MSH samples were collected twelve times over a period of 24 hours (0400h – 0700h – 0800h – 0900h – 1000h – 1200h – 1300h – 1400h – 1600h – 1900h – 2000h – 2400h) for the circadian study. Leptin, growth hormone (GH) and cortisol were assessed six times over the day (0800h – 1200h – 1600h –2000h – 2400h – 0400h). Samplings started at 0400h, after an overnight fasting at the hospital and IGF-1, 17β-oestradiol, free T3, SHBG, were measured at 08:00, before breakfast. Standardized meals were proposed at 8:15 (400 kcal), 12:15 (800 kcal) and 19:15 (800 kcal) and certainly eaten in CT and controls group. Snacks were not allowed in addition to the standardized meals. Food intake was not imposed or verified.

### Body composition measurements

Dual-energy x-ray absorptiometry (DXA) allowed the quantification of the percentage of total body fat mass and fat-free mass expressed in kilograms (LUNAR, DPX-L, <1%CV).

### Hormone assays

α-MSH concentrations in plasma samples was measured by a radioimmunoassay (RIA) set up in the laboratory as previously described [18]. Briefly, synthetic α-MSH (1 μg) was radiolabeled with 0.5 mCi Na ^125^I (Perkin-Elmer, Waltham, MA, USA) by means of the chloramine T method. Incubation was performed in 0.1 M phosphate buffer (pH 7.4) containing 0.1% BSA and 0.1% Triton X-100. The final dilution of the antiserum against α-MSH (# 812801) was 1: 30 000 and the total amount of tracer was 8000 cpm/tube. The production and characterization of the antibodies against α-MSH (generously provided by Dr MC Tonon, University of Rouen, France) has been previously described [18]. The incubation was carried out at 4°C for 48 h. Separation of the antibody-bound fraction was performed by adding 100 μl of bovine γ-globulins (1%) and 1 ml of polyethylene glycol 8000 (20%). The tubes were maintained 20 min at room temperature (21°C) and centrifuged (4000 g, 4°C, 30 min). The supernatants were removed and the precipitates containing the bound fraction were counted in a γ-counter (LKB Wallac, Rockville, MD, USA). The standard curves were set up with synthetic α-MSH at concentration ranging from 2 to 5000 pg/tube. The sensitivity threshold of the α-MSH RIA was 1 pg/tube.

Plasma NPY was evaluated by using an ELISA commercial kit (Hölzel Diagnostika, Köln, Germany) according to the manufacturer’s recommendations. Standard curves were performed with synthetic human NPY (concentration range 2.5–200 pg, sensitivity threshold—2 pg/tube).

Assessment of other hormonal parameters including IGF-1, 17β-oestradiol, free T3, SHBG, Leptin, growth hormone (GH) and cortisol and body composition measurements was previously described [6].

### Statistical analysis

All values are expressed as mean ± SEM. Intergroup comparison of one-time measured parameters was performed by ANOVA followed by a post-hoc test (Fisher’s Protected Least Significant Difference) when significant. Two-factor repeated measures ANOVA (group x time) followed by an adapted post-hoc analysis to evaluate inter- and intra-group differences was used for NPY and α-MSH variations. Point-by-point and 24-hour mean values correlations between NPY and α-MSH were calculated in each group of the present study and across the entire study samples. Correlations between 24-hour mean NPY or α-MSH levels and other antropometric and hormonal parameters were also evaluated. Statistical significance was set at p < 0.05. All statistical analyses were performed with the StatView 4.5 software (Abacus Concepts, Inc., Palo Alto, CA).

## Results

Antropometric and hormones characteristics of the three groups included in the study are presented in Table 1. Mean cortisol and GH, free T3, IGF I, estradiol, leptin and fat mass were significantly different in AN as compared to CT or to controls. Despite lower BMI (p<0.001) or fat mass and leptin (p<0.05) between CT and control groups, cortisol, GH, free T3, IGF I and estradiol remained in the same range.

**Table 1:** Antropometric and hormonal parameters in studied groups.

**Parameter**	**AN (n = 23)**	**CT (n = 22)**	**C (n = 14)**	p<0,05
**Age (yrs)**	22.5±1.3	23.2±0.5	22.6±1.6	-
**BMI (kg/m^2^)**	14.6±0.5	15.9±0.1	21.6±0.3	a, b
**Fat mass (kg)**	5.8±0.7	8.7±0.4	19.3±0.7	a, b, c
**Fat mass (%)**	15.3±1.6	22.3±1.0	32.9±1.3	a, b, c
**24 hours mean leptin (μg/L)**	1.9±0.1	6.4±0.3	14.0±0.6	a, b, c
**Free T3 (pmol/L)**	3.0±0.07	4.3±0.06	4.5±0.05	a, b
**17β-estradiol (ng/L)**	11.8±1.1	49.2±4.2	53.2±6.7	a, b
**IGF1 (μg/L)**	128±6	287±10	230±7	a, b
**24 hours mean GH (mUI/L)**	12.2±1.6	6.4±1.0	6.6±1.0	a, b
**24 hours mean cortisol (nmol/L)**	453±20	256±18	274±18	a, b

a) AN *vs* C; b) AN *vs* CT; c) C *vs* CT

**Fig 1:**
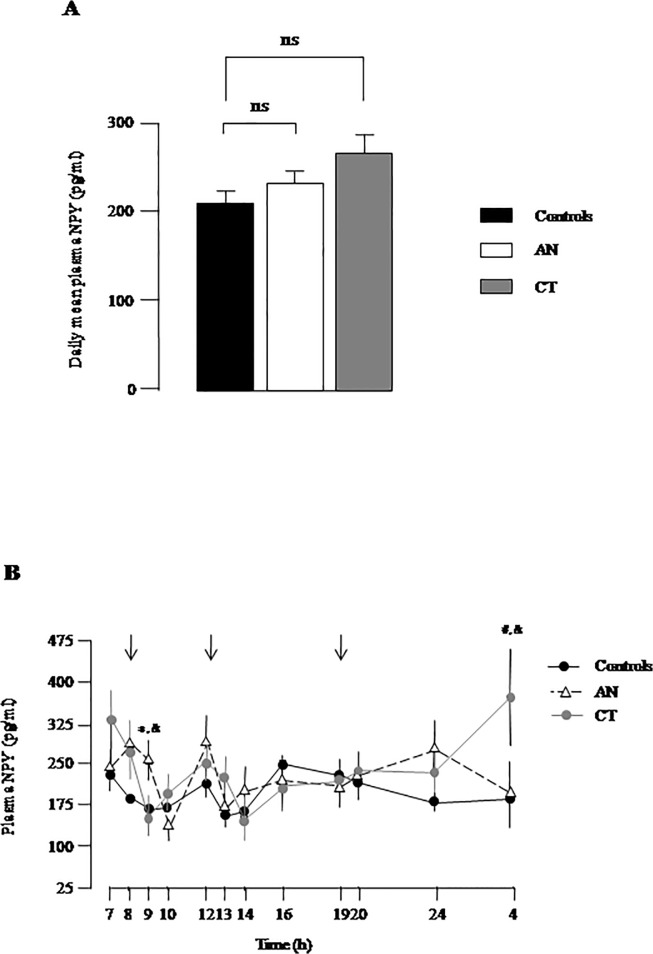
Plasma NPY levels in women with low body weight. (A) Daily mean values of plasma NPY levels in young women with anorexia nervosa (AN), constitutional thinness (CT) and in controls (Controls). Data represent means ± SEM of 4 independent experiments. ns, non significant. (B) Twelve-point circadian plasma levels of NPY in AN, CT and controls. The arrows indicate meal schedule. Each point represents mean ± SEM of 4 independent experiments. Significant point-by-point intergroup differences (p< 0.05): *, AN *vs* C; #, CT *vs* C; &, AN *vs* CT.

Daily mean NPY plasma levels, calculated as the mean of twelve time-points collected during 24-hour, was similar in AN, CT and controls (Fig. 1A). Repeated ANOVA measures of circadian profile of plasma NPY did not reveal any intergroup differences in average levels (group factor, F = 0.14, p = 0.8). By contrast, significant circadian variations of plasma NPY were observed in total group (time factor, F = 3.8, p<0.0001) with the occurrence of moderate peaks during the nychtemeron, at 0700–0800, 1200, and 1600 h (Fig. 1B). In addition, significant differences in circadian NPY variations were noticed among the three groups (group x time interaction, F = 2.3, p = 0.001). Specifically, higher levels of NPY were found in AN only at 0900h (p<0.01 *vs* C; p<0.03 *vs* CT). The lowest levels of NPY were detected after breakfast, (0900 in CT and C, 1000h in AN), and after lunch at 1300 h-1400 h in the three groups (Fig. 1B). In controls, a progressive decrease of NPY was observed in the evening and night whereas CT women displayed a NPY peak at 0400h (p<0.01 *vs* C; p<0.05 *vs* AN) (Fig. 1B).

**Fig 2:**
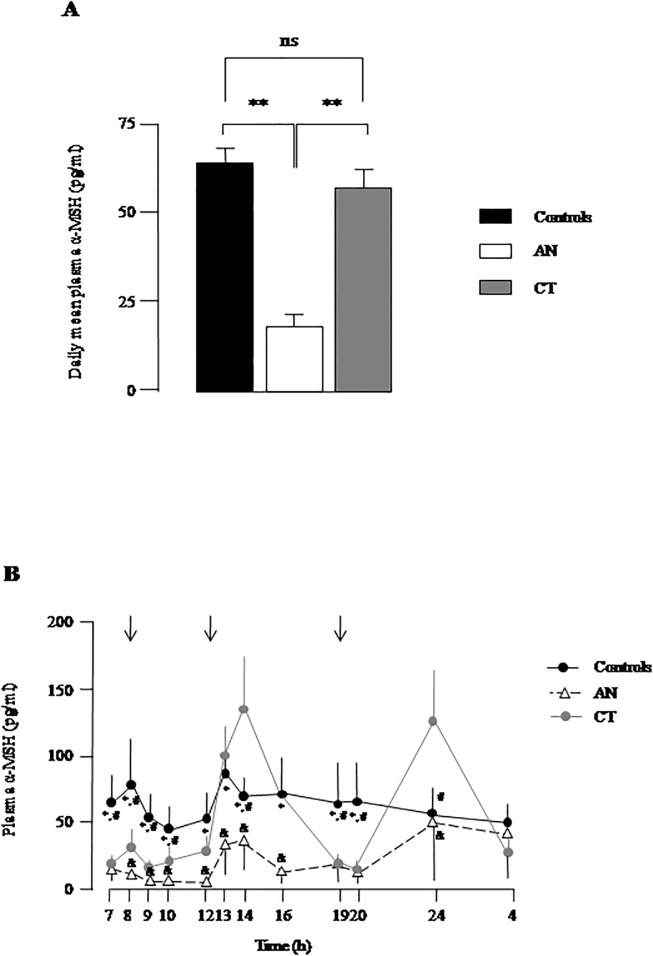
Plasma α-MSH levels in women with low body weight. (A) Daily mean values of plasma α-MSH levels in young women with anorexia nervosa (AN), constitutional thinness (CT) and in controls (Controls). Data represent means ± SEM of 4 independent experiments. ns, non significant; **, p<0.01. (B) Twelve-point circadian plasma levels of α-MSH in AN, CT and controls. The arrows indicate meal schedule. Each point represents mean ± SEM of 4 independent experiments. Significant point-by-point intergroup differences (p< 0.05): *, AN *vs* C; #, CT *vs* C; &, AN *vs* CT.

Daily mean plasma α-MSH levels were significantly lower (p<0.01) in AN as compared to controls and to CT (Fig. 2A). ANOVA analysis of α-MSH circadian profiles revealed significant intergroup differences in average levels (group factor, F = 4.2, p = 0.01). Differences in circadian α-MSH variations were found among the three groups (group x time interaction, F = 3.5, p = 0.03). In controls, plasma α-MSH levels presented moderate prandial peaks at breakfast (0800h) and lunch (1300–1400 h) but not at diner (Fig. 2B). Point-by-point comparisons revealed significantly lower α-MSH levels in AN *vs* C (p<0.05) all over the nychtemeron except at night (Fig. 2B). No significant prandial rise in α-MSH was noticed during the nycthemeron in AN (Fig. 2B). The circadian profile of plasma α-MSH in CT was different from controls and AN (Fig. 1B). In the morning, α-MSH levels in CT were significantly lower (p<0.05) than in controls with values between those of AN and controls (Fig. 1B). Then, CT women showed an important peak of circulating α-MSH after lunch (p<0.05 *vs* controls). α-MSH levels returned to basal values in the evening (at 1900 and 2000 h) and peaked again at 2400 h (p<0.05 *vs* C and AN). Despite these important circadian profile differences, the daily mean plasma α-MSH levels were similar in the CT and C groups (Fig. 2A).

No point-by-point correlation was found between NPY and α-MSH in any of the studied group or in total group of subjects. No correlation was noticed between NPY and α-MSH 24-hour mean values either. However, 24-hour mean values of α-MSH positively correlated with those of leptin (r = 0.54, p = 0.02) in total group. No intragroup correlation was found between NPY or α-MSH, and the other parameters.

## Discussion

This study investigates for the first time the circadian profiles of NPY and α-MSH in two populations of young women with low body weight, AN and CT.

The present data reveal that plasma NPY levels after an overnight fasting are similar in AN and CT as compared to controls. Fasting plasma NPY has previously been investigated in AN patients and the data are controversial. Indeed, some authors report decreased levels of plasma NPY in AN patients [8], or increased concentrations [9–12], or unchanged levels [13]. In our study, we found higher NPY levels in AN only 0900h (in the postprandial period) but not in fasting conditions. Overall, daily mean values showed that plasma NPY concentrations are unchanged in AN and CT patients as compared to healthy women. This finding contrasts with previous studies reporting that other orexigenic neuropeptides including ghrelin [3, 6], 26RFa [4] and orexins [19] are upregulated in AN patients, leading to the concept that AN subjects exhibit a global orexigenic profile in response to chronic undernutrition to restore energy balance [4]. Here we show that, in contrast to the other orexigenic neuropeptides, NPY is not up-regulated in AN, suggesting that the NPY system may play a role in the inability of anorectic women to adapt food intake to their energy demand.

To our knowledge, evolution of plasma α-MSH levels has never been investigated in AN. Here, we show for the first time that α-MSH levels are drastically decreased all over the day in AN patients (with the exception of the night) as compared to the control group. Consistent with this finding, a recent study reports a down-regulation of peripheral POMC mRNA in patients with AN [20]. Interestingly, the circadian profile of α-MSH in the AN group is very similar to that previously described for the other anorexigenic hormone leptin [2, 3] and, indeed, we found a positive correlation between circulating α-MSH and leptin. The circadian decrease of α-MSH levels in AN join the global adaptive appetite regulating profile of AN women, with a decrease of anorexigenic hormones and an increase of orexigenic peptides, necessary to promote energy intake in response to the negative energy balance of these patients. Interestingly, it has been recently reported that autoantibodies cross-reacting with α-MSH were elevated in AN patients raising the possibility that this increase in autoantibodies could be responsible, at least in part, for the low plasma α-MSH levels detected in anorexic subjects [21].

We have previously shown that CT women, which have an equivalent low BMI to AN subjects but no energy imbalance [15, 22], exhibit normal orexigenic peptides profile including ghrelin [2, 3] or 26RFa [4]. Plasma NPY in CT, with a circadian profile similar to that of control women, join this global panel, supporting the idea that the orexigenic pathway of appetite regulation is not altered in CT patients. In contrast, CT women have a particular circadian plasma α-MSH profile, with low levels in the morning and the afternoon that may contribute to their particular unrestricted feeding behavior we previously described [15]. This basal α-MSH tone is consistent with their leptin levels intermediate between AN and controls [3]. However, the important peak of α-MSH at mid-day prolonged until 1600h reveals that food intake is accompanied by an important satiety signal in CT women. Interestingly, we previously reported that PYY, another satiating peptide, was slightly increased in CTs [2]. This dramatic rise in α-MSH just after lunch may explain, at least in part, why these young women are rapidly food satisfied, and thus ingest low amounts of food and multiply their snacks. Indeed, we have previously observed high postprandial responses in PYY and total GLP-1 in CT women which were associated with smaller portioned-meals and more snacking [22]. In addition, CT women also show a dramatic rise of plasma α-MSH at midnight whose physiological relevance deserves further investigation.

One limitation of the present study concerns the source and the physiological relevance of circulating NPY and α-MSH. In the periphery, NPY is widely distributed in the sympathetic nerves, the adrenal medulla and the adipose tissue [23]. When injected peripherally, NPY increases body weight and stimulates accumulated fat mass [24, 25]. More specifically, NPY exerts hyperplasic, adipogenic and antilipolytic effects leading to increased energy storage in adipose tissue cells [23]. It is widely accepted that α-MSH is primarily, if not exclusively produced by the intermediate lobe of the pituitary in most of the mammals [26], indicating that the main source of circulating α-MSH is the pituitary gland. However, in human, only a vestige of the intermediate lobe is found, suggesting that α-MSH detected in the general circulation corresponds to a diffusion originating from the Arc [27]. Peripheral administration of α-MSH analogues results in acceleration of weight loss during a fast and increase of free fatty acid levels [27]. Collectively, these observations strongly suggest that, at the periphery, the NPY system is closely linked to positive energy balance whereas the α-MSH system mediates negative energy balance, as observed in the hypothalamus.

Another limitation was represented by the lack of data on the amount of food ingested by the AN subjects during the study. Standardized meals were proposed to all of the participants without any verification of food intake. The subjects were supposed to conserve their feeding behavior habits. The meals were certainly eaten by CT and controls. Although most of the AN subjects returned empty trays, we did not impose them to eat all of the food and we did not surveyed them during the meals. It was therefore difficult to evaluate the real amount of food ingested by the AN patients, and therefore to determine an eventual correlation with plasma NPY and α-MSH levels.

To conclude, in AN patients, the NPY system is not up-regulated by chronic undernutrition suggesting that this may play a role in the inability of anorectic women to adapt food intake to their energy demand. In contrast, the low circadian α-MSH levels observed in AN integrate the adaptive profile of appetite regulation of this disease. In CT women, an important peak of α-MSH is detected during lunchtime that could block food intake and explain why these patients are rapidly food satisfied.
